# AlphaMate: a program for optimizing selection, maintenance of diversity and mate allocation in breeding programs

**DOI:** 10.1093/bioinformatics/bty375

**Published:** 2018-05-02

**Authors:** Gregor Gorjanc, John M Hickey

**Affiliations:** The Roslin Institute, Royal Dick School of Veterinary Studies, The University of Edinburgh, Scotland, UK

## Abstract

**Summary:**

AlphaMate is a flexible program that optimizes selection, maintenance of genetic diversity and mate allocation in breeding programs. It can be used in animal and cross- and self-pollinating plant populations. These populations can be subject to selective breeding or conservation management. The problem is formulated as a multi-objective optimization of a valid mating plan that is solved with an evolutionary algorithm. A valid mating plan is defined by a combination of mating constraints (the number of matings, the maximal number of parents, the minimal/equal/maximal number of contributions per parent, or allowance for selfing) that are gender specific or generic. The optimization can maximize genetic gain, minimize group coancestry, minimize inbreeding of individual matings, or maximize genetic gain for a given increase in group coancestry or inbreeding. Users provide a list of candidate individuals with associated gender and selection criteria information (if applicable) and coancestry matrix. Selection criteria and coancestry matrix can be based on pedigree or genome-wide markers. Additional individual or mating specific information can be included to enrich optimization objectives. An example of rapid recurrent genomic selection in wheat demonstrates how AlphaMate can double the efficiency of converting genetic diversity into genetic gain compared to truncation selection. Another example demonstrates the use of genome editing to expand the gain-diversity frontier.

**Availability and implementation:**

Executable versions of AlphaMate for Windows, Mac and Linux platforms are available at http://www.AlphaGenes.roslin.ed.ac.uk/AlphaMate.

## 1 Introduction

This paper describes the AlphaMate program that optimizes selection, maintenance of genetic diversity and mate allocation in breeding programs. Breeding programs aim to achieve defined targets over the course of a time horizon. Some programs select individuals to improve future performance, while other programs try to maintain the current state or even save a population from extinction. In all cases optimal management of genetic diversity within the bounds of practical constraints is crucial to sustainably support the current and yet unknown future targets. For example, breeding programs that select for improved performance must balance short and long-term genetic gain by avoiding excessive use of elite individuals. While elite individuals increase the mean of next generations, their excessive use also significantly reduces the amount of genetic diversity. This reduction limits the potential for long-term improvement. Breeding programs that focus solely on maintenance of diversity must also ensure that individuals contribute in a somewhat balanced manner. Therefore, breeding programs must balance individuals’ contributions to future generations to ensure long-term viability.

The optimal contribution theory formulates balancing selection and maintenance of genetic diversity as optimization of individuals’ contributions to the next generation under constrained rate of group coancestry; see Woolliams *et al.* (2015) for review. Contributions can be optimized with two approaches. The first approach optimizes contributions to maximize genetic gain under a constrained rate of group coancestry amongst the contributors or to only minimize group coancestry. This optimization prevents the loss of genetic diversity above the accepted rate of coancestry. Optimization of contributions can be followed by mate allocation to minimize inbreeding of individual matings. This second optimization prevents excessive inbreeding depression in resulting progeny. These two optimizations can be solved with deterministic optimization methods that vary according to the mathematical formulation of the problem, e.g. Lagrangian multipliers (Meuwissen, 1997), linear programming (Toro and Pérez-Enciso, 1990), or quadratic programming (Pong-Wong and Woolliams, 2007). The second approach jointly optimizes contributions and mate allocations via optimization of a mating plan (Akdemir and Sánchez, 2016; Kinghorn and Shepherd, 1999). The joint optimization does not have an analytical form and has to be solved with stochastic or metaheuristic methods, such as evolutionary algorithms. These methods can easily accommodate constraints and multiple objectives in comparison to deterministic algorithms, but usually require more computing time.

Existing programs that implement the above described approaches are often applicable to specific applications and are not generically applicable to both animal and plant populations or do not accommodate application of modern biotechnologies such as genome editing. The aim of this work is to present a flexible program AlphaMate that covers all these use cases. We describe its methodology and show its application in two examples (i) maximizing efficiency of converting genetic diversity into genetic gain in a rapid recurrent genomic selection program for wheat and (ii) expanding the gain-diversity frontier with genome editing.

## 2 Materials and methods

AlphaMate by default jointly optimizes contributions and mate allocations. The goal of this optimization is to find a valid mating plan that delivers desired targets. This is achieved with an evolutionary optimization of a single objective or multiple objectives simultaneously.

A valid mating plan is defined by a combination of mating constraints: (i) the number of matings, (ii) the maximal number of parents, (iii) the minimal, equal, or maximal number of contributions per parent, or (iv) allowance for selfing.

The desired targets formulate optimization objectives, such as: (i) maximize genetic gain, (ii) minimize group coancestry amongst contributors, (iii) minimize expected inbreeding of individual matings, (iv) maximize genetic gain with constrained group coancestry or inbreeding, or (v) as (i) or (iv) but with the ability to genome edit a fixed set of contributors.

**Fig. 1.**
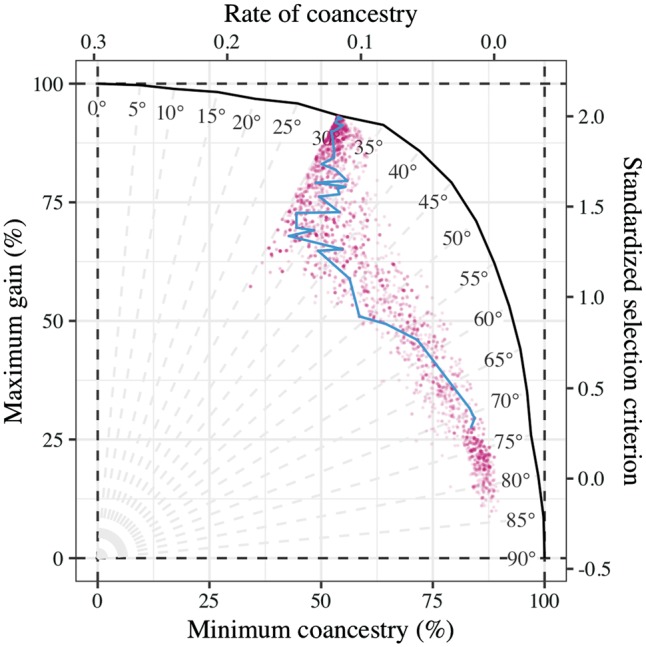
Trade-off between genetic gain and group coancestry and an optimization path of evolutionary algorithm (target set to 30°, dots show evaluated solutions, line shows evolution of the best solution)

Optimization is performed with an evolutionary algorithm based on differential evolution (Storn and Price, 1997) with modifications to avoid pre-mature convergence (Gondro and Kinghorn, 2009). For a single target, we optimize a single objective function accounting for mating constraints. For multiple targets, we perform multiple objective optimization in two steps; see e.g. Deb (2014) for review. First, we optimize single objective functions for each target separately to find bounds of the objective space and normalize objectives. Second, we use the ε–constraint method to either: (i) find a Pareto-optimal solution with targeted balance between objectives or (ii) evaluate the whole frontier of Pareto-optimal solutions. A Pareto-optimal solution is the best solution with a specific balance between objectives. The Pareto frontier is a set of optimal solutions, which are useful when a breeder does not have clearly defined targets and can explore solutions with different balance between targets to reach a decision. Figure 1 demonstrates the Pareto frontier of genetic gain and group coancestry and the optimization path of a solution.

Optimization works with mating plans, which we encode as proposed by Kinghorn and Shepherd (1999). We ensure that mating plans are valid in two ways. First, we fix solutions, e.g. we trim contributions to user defined limits and round them to integer values (Lampinen and Zelinka, 1999). Second, when fixing is not sufficient, we penalize invalid mating plans so that the evolutionary algorithm advances (more) valid mating plans.

## 3 Use

AlphaMate was written in object oriented Fortran 95 as a standalone program and compiled versions are available for Windows, Linux, and Mac platforms. A single specification file controls the program. In this file, a user specifies: (i) input files, (ii) mating constraints, (iii) desired targets and (iv) optimization controls. Below we describe these groups of specifications, while the full list is available in the AlphaMate manual.

- The basic files are the coancestry matrix, selection criteria and gender information for candidates. The coancestry matrix and selection criteria can be based on pedigree or genomic data. Additionally, further individual or mating specific information can be provided to enrich optimization objectives.
- Mating constraints can be gender specific or generic to accommodate different reproductive systems. A user can specify all the mating constraints or a subset of them depending on the optimization objectives and biologic or logistic reasons.
- Desired targets define the optimization objectives. For ease of use, we allow for various forms of some targets, e.g. constraint on the loss of genetic diversity can be specified with the targeted value of coancestry, rate of coancestry, percentage of the minimum possible coancestry, or trigonometric degrees between genetic gain and group coancestry (Fig. 1).
- Optimization controls specify weights to combine multiple targets into a single objective function or to penalize invalid mating plans, and parameters of evolutionary algorithm such as the number of iterations, convergence criteria, etc.

The AlphaMate output consists of: (i) input data summary, (ii) list of contributors with associated data, (iii) optimized mating plan, (iv) optimization log and (v) the seed value for random number generator to enable reproducibility. A utility R script is provided to plot the Pareto frontier and the optimization paths.

## 4 Demonstration

**Fig. 2.**
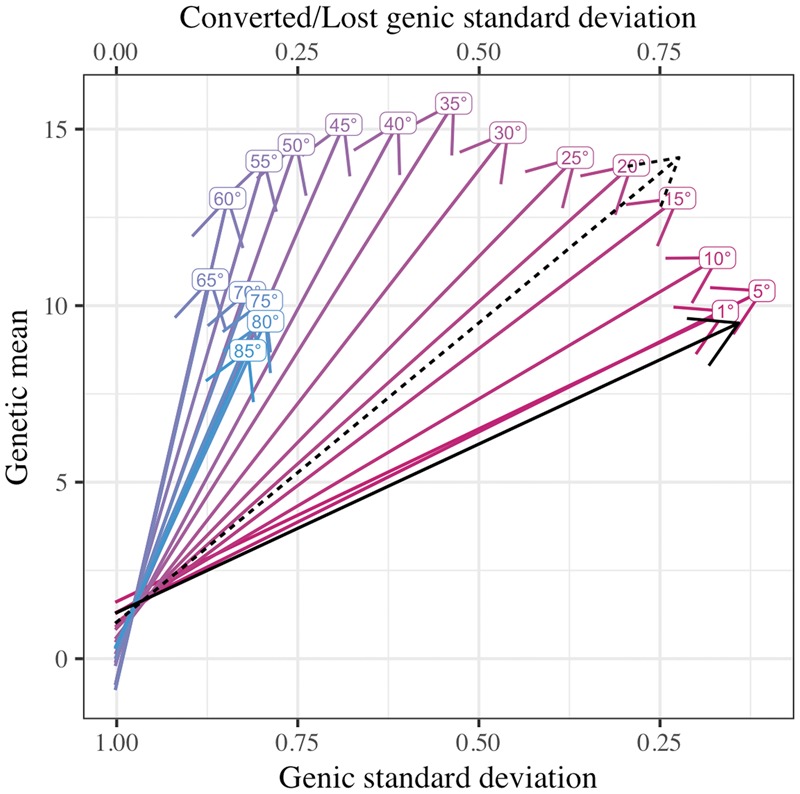
The genetic mean and genic standard deviation over 20 years of a wheat breeding program optimized with AlphaMate for different balance between selection and maintenance of genetic diversity defined by trigonometric degrees; black lines denote truncation selection with one (dashed) or four (full) crosses per parent

We demonstrate the use of AlphaMate with two examples. The first example optimizes conversion of genetic diversity into genetic gain based on a subset of the results from a previous study we undertook to model the benefit of rapid recurrent genomic selection in wheat (Gorjanc *et al.*, 2017). Here, we compare AlphaMate to truncation selection method over 20 years with four recurrent selection cycles per year with 10 simulation replicates. In each cycle, we used a pool of 32 parents to generate 16 crosses with 160 progeny in total. We used AlphaMate to optimize selection and mate allocation with a constraint that a parent could contribute up to four crosses. We supplied AlphaMate with genomic estimates of breeding values and a genomic coancestry matrix that measured the proportion of marker alleles in common between the progeny. We ran ten simulations, collected genetic mean and genic standard deviation in progeny for every cycle and fitted linear regression on this data. In Figure 2, we show the evolution of genetic mean and genic standard deviation over the 20 years as influenced by different balance between selection and maintenance of genetic diversity achieved via different trigonometric degrees. We also show results for the truncation selection method, where we ignored maintenance of genetic diversity and parents either contributed one or four crosses. There is a clear effect of balancing the two objectives on the long-term performance of the breeding program. In comparison to truncation selection with one (four) cross per parent AlphaMate with the target of 35° delivered 65% (11%) higher genetic gain with 278% (139%) lower reduction of genic standard deviation, which translates to a 242% (93%) higher efficiency of converting genetic diversity into genetic gain. We note that truncation selection with one cross per parent achieved slightly higher genetic gain than AlphaMate with comparable efficiency (15–20°), which suggests that group coancestry based on the proportion of shared marker alleles might not be the best metric for the long-term maintenance of genetic diversity in populations under selection. This is subject of our future research.

**Fig. 3.**
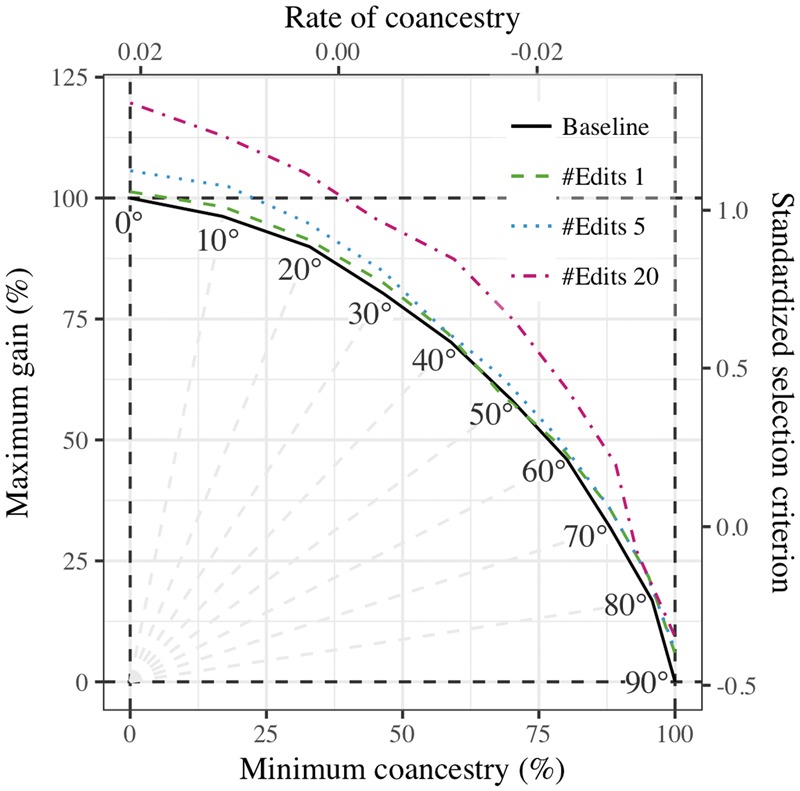
Trade-off between genetic gain and group coancestry and its modification with genome editing

The second example expands the gain-diversity frontier based on our previous modelling of the genome editing potential to improve quantitative traits along standard selection methods (Jenko *et al.*, 2015). By way of example genome editing could improve the genetic merit of the top individuals or the average individuals. If used optimally, the latter option might have the potential to expand the gain-diversity frontier, i.e. expand the Pareto frontier of genetic gain and group coancestry. To test this, we have simulated one replicate of a breeding program as in Jenko *et al.* (2015) with 1000 selection candidates out of which we aimed to select 25 males and all 500 females with equalized contributions. In addition, we assumed to have resources to genome edit any 5 males, each at 1, 5, or 20 top causal loci. The question in such a setting is, which males should be selected and edited to maximize genetic gain for a given increase in group coancestry. We evaluated this by first calculating the genetic merit that male candidates could have been achieved with editing. We then provided the non-edited and edited genetic merit of the candidates to AlphaMate and jointly optimized which males should be selected and edited. To this end we have added to optimization a set of ‘edit rank’ variables of length equal to the number of candidates for editing. When calculating the genetic gain, we used ‘edited’ genetic merit for individuals with the highest ‘edit rank’ and ‘non-edited’ genetic merit for the others. In Figure 3, we show the Pareto frontier without and with genome editing. The results show that genome editing expanded the frontier. However, the expansion was substantially only when we edited 20 top causal loci and when target was not solely on minimum coancestry. At 30° the baseline maximum gain was 80% and the baseline minimum coancestry was 46%. With editing 5 or 20 loci the maximum gain improved to, respectively, 85% or 96%, while the minimum coancestry only slightly deteriorated to 45%.

## 5 Conclusion

In this paper, we have described the AlphaMate program that optimizes selection, maintenance of diversity and mate allocation in breeding programs. The program enables both animal and plant breeding programs to be more optimal and facilitates new research opportunities.

## Funding

This work was supported by the Biotechnology and Biological Sciences Research Council (GB) [BBS/E/D/30002275, BB/N015339/1, BB/L020467/1, BB/M009254/1].

*Conflict of Interest*: none declared.

## References

[bty375-B1] Akdemir D., Sánchez J.I. (2016) Efficient breeding by genomic mating. Front. Genet., 7, 210. 27965707 10.3389/fgene.2016.00210 PMC5126051

[bty375-B2] Deb K. (2014) Multi-objective optimization In: Search Methodologies, Springer, pp. 403–449.

[bty375-B3] Gorjanc G. et al (2017) Optimal cross selection for long-term genetic gain in two-part programs with rapid recurrent genomic selection. doi: 10.1101/227215. 10.1007/s00122-018-3125-3 PMC6096640 29876589

[bty375-B4] Gondro C., Kinghorn B.P. (2009) Application of Evolutionary Algorithms to Solve Complex Problems in Quantitative Genetics and Bioinformatics. University of Guelph, Guelph, Canada, p. 96.

[bty375-B5] Jenko J. et al (2015) Potential of promotion of alleles by genome editing to improve quantitative traits in livestock breeding programs. Genet. Sel. Evol., 47, 55. 26133579 10.1186/s12711-015-0135-3 PMC4487592

[bty375-B6] Kinghorn B.P., Shepherd R.K. (1999) Mate selection for the tactical implementation of breeding programs. Assoc. Advmt. Anim. Breed. Genet., 13, 130–133.

[bty375-B7] Lampinen J., Zelinka I. (1999) Mixed variable non-linear optimization by differential evolution. Proc. Nostradamus, 99, 7–8.

[bty375-B8] Meuwissen T.H.E. (1997) Maximizing the response of selection with a pre-defined rate of inbreeding. J. Anim. Sci., 75, 934–940. 9110204 10.2527/1997.754934x

[bty375-B9] Pong-Wong R., Woolliams J.A. (2007) Optimisation of contribution of candidate parents to maximise genetic gain and restricting inbreeding using semidefinite programming. Genet. Sel. Evol., 39, 3–25. 17212945 10.1186/1297-9686-39-1-3 PMC2739432

[bty375-B10] Storn R., Price K. (1997) Differential evolution–a simple and efficient heuristic for global optimization over continuous spaces. J. Global Optim., 11, 341–359.

[bty375-B11] Toro M., Pérez-Enciso M. (1990) Optimization of selection response under restricted inbreeding. Genet. Sel. Evol., 22, 93–107.

[bty375-B12] Woolliams J.A. et al (2015) Genetic contributions and their optimization. J. Anim. Breed. Genet., 132, 89–99. 25823835 10.1111/jbg.12148

